# Prevention and intervention against obesity and overweight in the military: a systematic review

**DOI:** 10.1186/s12995-025-00480-7

**Published:** 2025-10-07

**Authors:** Lorenz Scheit, Lisa Baustert, Jan Schröder

**Affiliations:** 1 Department I–Internal Medicine, Bundeswehr Hospital Hamburg, Lesserstr. 180, Hamburg, 22049 Germany; 2 Department of Sports Medicine, Faculty for Psychology and Human Movement Science, Institute for Human Movement Science, University of Hamburg, Turmweg 2, Hamburg, 20148 Germany

**Keywords:** Obesity, Overweight, Prevention, Military, Waist circumference, BMI

## Abstract

**Background:**

Overweight and obesity have a negative impact on health and have a detrimental effect on the operational readiness of soldiers. Different prevention and intervention measures against obesity include diet, physical activity, education, coaching or medication or a combination of several aspects have been investigated. This review systematically assesses the effectiveness of lifestyle, dietary, educational, and pharmacological interventions on weight, body composition, and military readiness in active-duty personnel.

**Methods:**

We carried out a systematic review based on the Preferred Reporting Items for Systematic Reviews and Meta-Analyses (PRISMA) guidelines. A search was conducted in various electronic databases from 2000 to July 2024. The search strategy combined three concepts: military population, outcome terms, interventions or programs. For the review, 21 articles met the inclusion criteria (total n = 1696). Where possible, the effect size (ES) was calculated.

**Results:**

In studies that only examined exercise, minor effects were reported, e.g. a reduction in weight (-0.5 kg), Body Mass Index (BMI) (-0.3 kg/m²), waist circumference (-0.1 cm) and body fat percentage (-1.1%) without relevant statistical ES. Studies with nutritional programs reported low to moderate ES. Combined programs including dietary and exercise countermeasures showed to be more effective than programs based on diet or exercise alone. Combined programs with exercise and educational methods showed a moderate to large effect size (ES 0.6–1.3) for weight reduction. Pharmacological treatment for reducing fat intake resulted in a larger effect sizes for weight loss. The greatest efficacy (ES > 1.0) was observed for a combined intervention program consisting of lifestyle changing components based on individually tailored cognitive behavioral therapies, psychoeducation, exercise and nutritional interventions.

**Conclusions:**

Effective countermeasure for reducing body weight found in this study were combined interventions, like education on lifestyle changes, dietary habits and promotion of physical activity in military personnel, as well as by ketogenic dietary interventions combined with physical activity and followed by pharmacological intervention approaches. Combined interventions appear promising in some studies, but future evaluations may focus on combinations of physical activity and exercise with new pharmaceutical approaches like Semaglutide or Bimagrumab medication in the long term for military personnel due to probable favorable body composition adaptations and military readiness.

**Supplementary information:**

The online version contains supplementary material available at 10.1186/s12995-025-00480-7.

## Introduction

The steadily increasing prevalence of overweight and obesity in European and other industrialized nations over the past few years represents a growing challenge not only for healthcare systems. In 2007, it was shown that more than 50% of the European population was already overweight or obese [1]. In Germany, the proportion of obesity rose from 16.2 to 22.5% in males and from 16.2 to 23.3% in females between 1985 and 2002 [2]. This development in society as a whole does not stop at the professional group of soldiers. A recent prevalence study of more than 40,000 soldiers in the German Armed Forces from 2018 to 2022 showed that 18% of all soldiers were obese [3]. It is particularly important for soldiers to have a high level of mental and physical health and physical fitness due to their professional role of combat participants. Good physical fitness is the basic prerequisite for the efficient operational readiness of armed forces. Increasing tendencies towards obesity have an opposite impact. Irrespective of the relevance of high physical readiness, obesity and overweight are independent risk factors for metabolic and cardio-circulatory diseases. Current data show that cardiometabolic risk doubles in obese grade 1 people (BMI > 30) and even increases tenfold in obese grade 2 people (BMI > 35) [4]. Prevention and intervention measures to prevent or reduce overweight and obesity refer to very different strategies such as various diets, physical activities (strength training, aerobic training or high-intensity interval training - HIIT), behavioral intervention or drug treatment [5–8]. Successful prevention and intervention measures, especially for the relevant occupational group of soldiers, are in the focus of international research, as the relevance for military readiness is of great importance for social security. To the best of the authors’ knowledge, a systematic survey of prevention and intervention measures against overweight and obesity in the specific environment of military personnel has not yet been carried out. This systematic review examines the results of international prevention and intervention studies on overweight and obesity in soldiers. The aim of this study is to record previously investigated prevention and intervention measures against obesity and to assess the effect of these measures on body composition and fitness outcomes in military personnel. Based on these findings, successful intervention programs for military personnel can be further adopted and developed to reduce or stop the trend of increasing prevalence of obesity hampering military readiness.

## Materials and methods

This systematic review was conducted on the basis of the Preferred Reporting Items for Systematic Reviews and Meta-Analyses (PRISMA) guidelines [9]. A PICO scheme (population, intervention, control, outcome) was created to present the key concepts of the topic and to structure the literature search. The review was registered at PROSPERO review database in accordance with the PRIMSA 2020 checklist in a timely manner on 15 December 2024, prior to the start of data analysis (Registration Number: CRD42024622560). This systematic review was not created as a meta-analysis, due to the heterogeneity of the studies in study designs, populations, interventions, and outcomes (e.g., BMI vs. waist circumference, or the rate of 5% body weight loss), as well as due to a lack of systematically reported standard deviations or confidence intervals in absolute changes or correspondingly reported effect sizes.

### Search strategy and inclusion criteria

An electronic search was conducted on 16th July 2024 in four online databases (CINAHL, Livio, PubMed and Web of Science). It included studies from the beginning of 2000 to July 2024. The search strategy combined three concepts: [1] military population [2], outcome terms [3], interventions or programs. The search strategy adapted in each data base were reviewed by all authors and is shown in Table 1. There was no restriction regarding the language. The search results were managed with a bibliographic tool (Zotero).

**Table 1:** Search strategy and identified sources

Data Base	Key Words	Results
CINAHL	(“Military Personnel“[MeSH Terms] OR “armed forces” OR “army” OR “Bundeswehr”) AND (“Intervention” OR “prevention” OR “counter measures” OR “diet therapy“[MeSH Terms] OR “exercise“[MeSH Terms]) AND (“Overweight“[MeSH Terms] OR “obesity” OR “Body Weight” OR “BMI”) NOT (pregnant)	45
LIVIO	(“Military Personnel“[MeSH Terms] OR “armed forces” OR “army” OR “Bundeswehr”) AND (“Intervention” OR “prevention” OR “counter measures” OR “diet therapy“[MeSH Terms] OR “exercise“[MeSH Terms]) AND (“Overweight“[MeSH Terms] OR “obesity” OR “Body Weight” OR “BMI”) NOT (pregnant)	129
PubMed	(“Military Personnel“[MeSH Terms] OR “armed forces” OR “army” OR “Bundeswehr”) AND (“Intervention” OR “prevention” OR “counter measures” OR “diet therapy“[MeSH Terms] OR “exercise“[MeSH Terms]) AND (“Overweight“[MeSH Terms] OR “obesity” OR “Body Weight” OR “BMI”) NOT (pregnant)	897
Web of Science	ALL=((“Military Personnel“[MeSH Terms] OR “armed forces” OR “army” OR “Bundeswehr”) AND (“Intervention” OR “prevention” OR “counter measures” OR “diet therapy“[MeSH Terms] OR “exercise“[MeSH Terms]) AND (“Overweight“[MeSH Terms] OR “obesity” OR “Body Weight” OR “BMI”) NOT (pregnant))	625

### Selection process

**Fig. 1:**
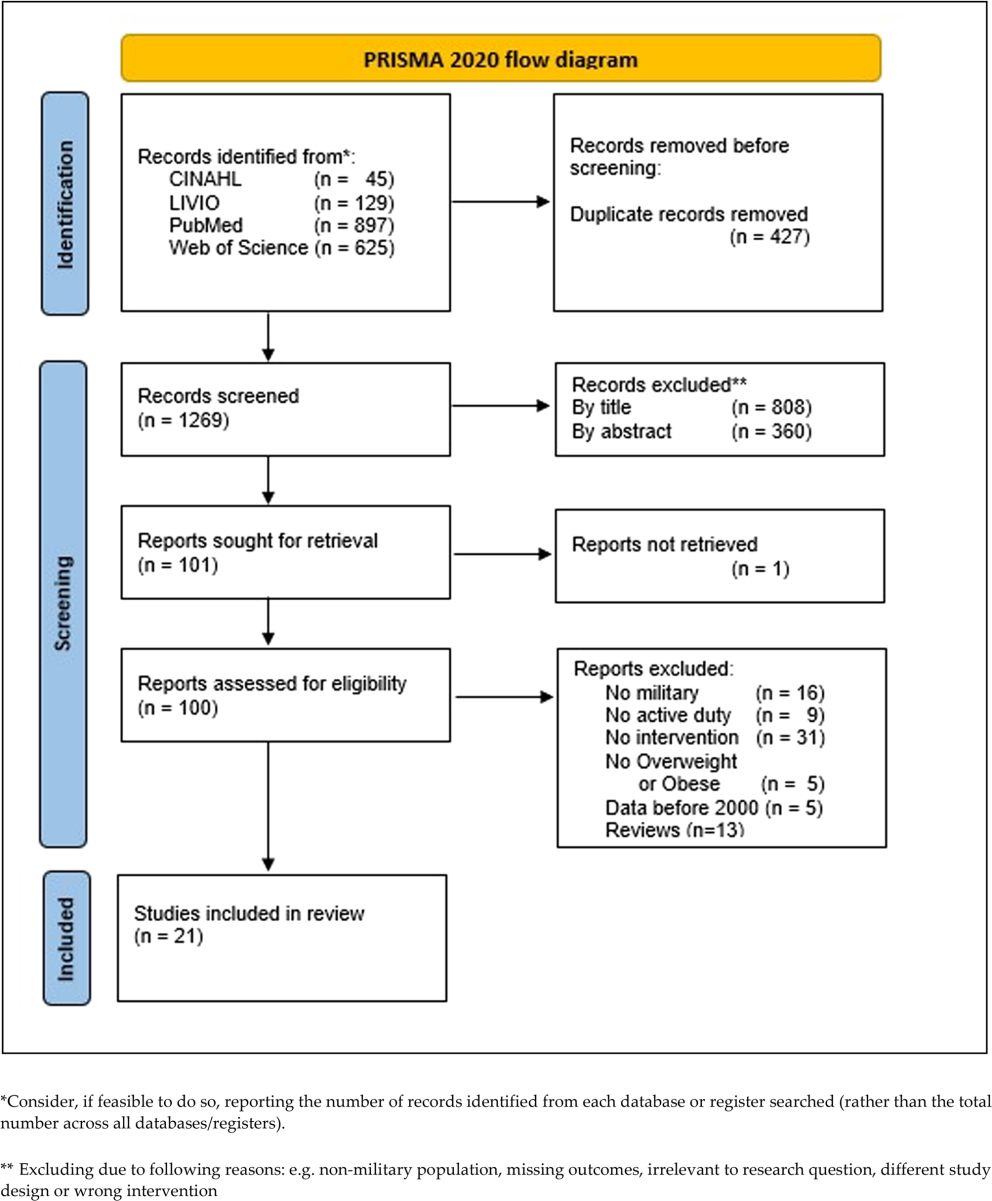
PRISMA 2020 flow diagram [9]

The data was further analyzed in accordance with the PRISMA flow-diagram (Figure 1). The search yielded 1696 studies. The first step was to remove the duplicates. Screening was then carried out first by title, then by abstract and finally by evaluation of the full texts.

Based on the previously created PICO scheme, the inclusion of studies was carried out according to the following points:

- Population: Studies were included if all participants were active military personnel over 18 years of age or if the results of these were reported separately from others. Studies with veterans, family members of soldiers or civilians were excluded. In addition, studies with pregnant women or postpartum mothers were excluded.
- Intervention: All countermeasures, both weight gain prevention and weight loss interventions, were included.
- Control: Studies were included both with and without a control group.
- Outcome: Studies were included, if intervention related changes of any body-constitution parameters were reported such as body weight, body fat or body mass index.

### Risk of bias

To assess the quality assurance, the 2013 National Heart, Lung, and Blood Institute (NHLBI) Quality Assessment Tool for Observational Cohort and Cross-Sectional Studies (https://www.nhlbi.nih.gov/healthtopics/study-quality-assessment-tools, accessed on 30 April 2025) was used [9]. The tool was generated by the NHLBI to check the internal validity of the proven studies. By answering 14 questions, the reviewer identifies a possible risk of bias. The quality score determined was used to classify the study quality according to the NHLBI Quality Rating” (Good, Fair, or Poor) with a maximum of 14 points that may be achieved here.

The PEDro-Scale was used to determine the risk of bias for randomized controlled trials. It consists of 11 items of which 10 items are used to classify the PEDro score. Total PEDro scores of 0–3 are considered ‘poor’, 4–5 ‘fair’, 6–8 ‘good’, and 9–10 ‘excellent [10].

### Content data analysis

In order to structure the effectiveness of the various approaches, we tried to extract the original effect sizes of the respective interventions, in the case they were given by the authors. If not, we tried to calculate effect sizes retrospectively from reported means and standard deviations or mean changes e.g. pre-post-counts, if applicable, using an Excel macro leading to Cohen’s d [11].

In general, effect sizes for mean differences of two groups are deemed to demonstrate small (≥ 0.2), moderate (≥ 0.5) or large (≥ 0.8) effects [12].

The studies showed different types of intervention, leading us to form groups according to corresponding main topics such as diet, exercise and medication, or combinations of these categories.

## Results

### Search strategy

Our systematic research identified 1269 original articles of which 21 studies were deemed to be suitable according to our inclusion criteria. For detailed reasons for any study exclusion see the PRISMA flow-chart illustration (Fig. 1). An overview about the evaluated studies in this review can be found in Table 2.

**Table 2:** Summary of evaluated studies

Interven-tion type	Study	Population	Duration	Absolute Change (AC)	Effect size (ES)	Risk of bias
Exercise (approx. 16 h/week intens. exercising)	Ceder-berg (2011) [13]	Finland *n* = 1476 (conscripts, 100% males) age 19.3 (19–28) BMI 23.9 (16.3–46) weight 75.1 (47.2–140.0) kg	52 weeks (follow-up)	weight − 0.4 (± 5.2) kg BMI − 0.3 (± 1.7) waist − 0.10 (± 5.8) cm BF% −1.1 (± 4.6)	ES not reported (not calculable)	NHLBI 11/14
Exercise (basic military training)	Vantarakis et al. (2022) [14]	Greece *n* = 185 (82.7% males) Conscripts, Navy freshmen BMI 23.2 (± 2.6) weight 72.6 (± 9.1)	10 weeks (94 intense sessions)	AC not reported ^#^ weight − 1.8 kg BMI − 0.6 BF% −1.4%	ES not reported (calculated as: weight 1.29 BMI 1.30 BF% 0.60, moderate to large)	NHLBI 9/14
Diet (low carb vs. low fat)	Zinn et al. (2017) [15]	New Zeeland *n* = 41 (76% males) BMI Ø 30 kg/m² age Ø 40 low carb *n* = 21 low fat *n* = 20	12 weeks	AC not reported ^#^ low carb (*n* = 14): weight − 5.3 kg waist − 4.8 cm low fat (*n* = 12): weight − 2.0 kg waist − 3.3 cm	favoring low carb: weight 0.39 waist 0.21 (small)	PEDro 5/11
Diet (synbiotic suppl.)	Parastouei (2022) [16]	Iran *n* = 60 (58% males) verum (*n* = 30) age 42.3 (± 1.5) BMI 32.5 (± 0.9) placebo (*n* = 30) age 40.6 (± 1.1) BMI 31.6 (± 0.8)	8 weeks	AC not reported ^#^ verum: BMI − 0.6 placebo: BMI + 0.1	BMI eta²p 0.001 for inter-action (trivial)	PEDro 8/11
Life-Style-Coaching (LIFE* program)	Bowles (2006) [17]	USA n (starters) = 93 (59.1% males) n (completers) = 53 (63.6% males) BMI 31.9 (± 2.7) (males) BMI 29.9 (± 3.5) (females)	52 weeks	AC not reported ^#^ BMI − 1.5 (males) weight ≈ −4.8 kg (males) BMI − 2.4 (females) weight ≈ −6.3 kg (females)	ES not reported (calculated as 1.18–1.39, large)	NHLBI 8/14
Life-Style-Coaching (BUND^$^)	Brandes (2007) [18]	Germany *n* = 11 (Bundeswehr obesity intervention program, course ST 010)	24 months	weight − 4.3 kg BMI − 1.3 waist − 3.7 cm SD not reported	ES not reported (not calculable)	NHLBI 10/14
Life-Style-Coaching (internet use)	Hunter (2008) [19]	USA Total *n* = 446 (50% males) age EXP 33.5 (± 7.4) Usual care (CON) 34.4 (± 7.2)	21 weeks	EXP weight − 1.3 (± 4.1) kg BMI − 0.5 (± 1.4) waist − 2.1 (± 4.3) cm ≥ 5% body weight loss: 22.6%	ES not reported (not calculable)	PEDro 7/11
Life-Style-Coaching (energy uptake diaries)	Shay et al. (2009) [20]	USA *n* = 39 after drop-out (59% males) age 35.3(± 9.3) BMI 33.0 (± 3.4) waist ≈ 104.1 (± 11.2) cm body fat% (BF%) 34.9 (± 7.3)	12 weeks after ShipShape	no differences between diaries weight − 2,8 (± 3.9) kg waist ≈ −5.6 (± 5.6) cm BF% −1.6 (± 2.6)	ES not reported (not calculable)	PEDro 6/11
Life-Style-Coaching (web site planners)	Stewart et al. (2011) [21]	USA *n* = 2417 (68.8% males) males (*n* = 1473) age 32.3 (± 8.1) BMI 28.9 (± 3.7) females (*n* = 755) age 30.9 (± 8.5) BMI 26.8 (± 4.0)	approx. 150 weeks Phase 1: 25 months Phase 2: 37 months	AC not reported ≥ 5% body weight loss: 12%	ES not reported (not calculable)	NHLBI 8/14
Life-Style-Coaching (behavior class 4 h “Be Well”)	Webber et al. (2012) [22]	USA *n* = 276 (77% males) Air-Force members age 19–51 (29 ± 6.9) BMI 27.9 (± 3.9) (males) BMI 25.2 (± 3.3) (females)	≈ 11 weeks (1–29)	AC not reported ^#^ BMI − 0.2 (males) waist 0.75 cm (males) BMI − 0.2 (females) waist 0.25 cm (females)	ES not reported (calculated as < 0.1, trivial)	NHLBI 10/14
Life-Style-Coaching (BUND^$^)	Sammito (2012) [23]	Germany inhouse: *n* = 425 BMI 33.7 (± 3.6) weight 108.6 (± 14.1) out-patient: *n* = 625 BMI 33.8 (± 4.2) weight 109.3 (± 15.6)	24 months (measures 12 months due to 60% drop out)	weight − 4.4 kg (inhouse) weight − 7.0 kg (out-patient) SD not reported ≥ 5% body weight loss: 28,7%	ES not reported (not calculable)	NHLBI 6/14
Life-Style-Coaching (BUND^$^)	Sammito (2013) [24]	Germany *n* = 665 age 40 (± 9.4) BMI 33.8 (± 4.2) weight 109.8 (± 16.5)	24 months (measures 12 months due to 80% drop out)	AC not reported ^#^ weight − 3.1 kg BMI − 0.9 waist − 3.1 cm ≥ 5% body weight loss: 12,2%	ES not reported (not calculable)	NHLBI 10/14
Life-Style-Coaching (BUND^$^)	Sammito (2016) [25]	Germany *n* = 334 age 43.5 (± 6.9) BMI 33.4 (± 3.6) weight 107.4 (± 13.8) kg	24 months	BMI − 1.0 (± 2.0) weight − 3.4 (± 6.6) kg waist − 3.8 (± 6.4) cm ≥ 5% body weight loss: 27,5%	ES not reported (not calculable)	NHLBI 9/14
Life-Style-Coaching (distance based, counselor vs. self-paced)	Krukowski et al. (2018) [26]	USA *n* = 248 (49.2% males) age 34.6 (± 7.5) weight 88 kg (± 14.3) overweight 43.5% obesity 56.5%	52 weeks	Counselor initiated: weight − 1.9 kg (± 4.1) waist − 2.7 cm (± 6.5) ≥ 5% body weight loss: 29.5% Self-paced: weight − 0.1 kg (± 3.8) waist − 1.7 cm (± 8.1) ≥ 5% body weight loss: 15.6%	ES not reported (not calculable)	PEDro 5/11
Life-Style-Coaching (PA pro-motion, 7 sessions)	Sanaeina-sab et al. (2020) [27]	Iran *n* = 84 (100% males) age 31.0 (± 7) BMI 29.0 (± 3.5)	12 weeks	AC not reported ^#^ BMI: EXP − 1.0 vs. CON − 0.3	EXP-BMI 0.54 (moderate)	PEDro 6/11
Life-Style-Coaching (mHealth, SAM use)	Gorny et al. (2022) [28]	Singapore *n* = 167 (100% males) completers *n* = 29 (18%) age 21–25	21 weeks following weight loss program	preliminary weight- loss after program: −15.6 (± 4.1) kg weight re-gain after: +5.3 (± 4.5) kg	ES not reported (not calculable)	NHLBI 8/14
combined programs: Life-Style-Coaching (non-pers.) & low intens. PA	Robbins et al. (2006) [29]	USA *n* = 68,591 BMI 24.0–29.9 EXP *n* = 3,502 (87% males) age 31.0 CON *n* = 65,089 (90% males) age 30.4	52 weeks	weight-loss Women: EXP ≈ −0.01 kg CON ≈ + 0.36 kg Senior Airmen: EXP ≈ −0.32 kg CON ≈ + 0.27 kg all other males EXP ≈ + 1.3 kg CON: ≈ +1.1 kg SD not reported	ES not reported (not calculable)	NHLBI 9/14
combined: Life-Style-Coaching & diet (meal-replace.)	Smith et al. (2010) [30]	USA *n* = 113 (67.3% males) EDU alone *n* = 56 BMI 33.1 (± 2.9) meal-replacement & EDU *n* = 57 BMI 33.1 (± 3.0)		EDU alone BMI − 1.1 (± 1.1) weight − 0.8 (± 2.6) kg meal-replacement BMI − 1.2 (± 1.0) weight − 2.0 (± 3.2) kg	ES not reported (calculated as 0.21–0.24, small)	PEDro 5/11
combined: Life-Style-Coaching & pharma-ceuticals (Orlistat)	Smith et al. (2012) [31]	USA *n* = 435 (75% males) completers: Orlistat & EDU *n* = 35 BMI 33.2 (± 3.1) placebo & EDU *n* = 22 BMI 33.5 (± 3.8)		Orlistat BMI − 1.1 (± 1.7) weight − 3.1 (± 4.7) kg placebo BMI − 1.0 (± 1.7) weight − 3.1 (± 5.2) kg	ES not reported (calculated as weight 0.19 BMI 0.33, small)	PEDro 6/11
combined programs: Life-Style-Coaching & diet (herbal suppl.) following MOVE! ^§^	McCarthy et al. (2017) [32]	USA *n* = 435 (73.4% males) age 30 (± 8)	12 weeks	Coaching groups: weight − 2,8 kg BMI − 0.65 waist − 1.3 cm BF% −1.0% SD not reported	weight 0.07 BMI 0.10 waist 0.06 BF% 0.11 (trivial)	PEDro 6/11
combined programs: exercise & diet (keto-genic vs. mixed)	LaFountain et al. (2019) [33]	Finland *n* = 29 after drop-out (86.2% males) ketogenic age 27.4 (± 6.8) BMI 27.9 (± 2.9) mixed diet age 26.4 (± 9.0) BMI 24.9 (± 2.4)	12 weeks	Ketogenic diet (*n* = 15): weight − 7,7 kg BF% −5.1 mixed diet (*n* = 14): weight − 0,1 kg BF% −0.7 SD not reported	ES not reported (not calculable)	NHLBI 10/14

*AC* Absolute changes, *BMI* Body mass index (kg/m²), *BF%* Body fat percentage, *BUND*^$^ Bundeswehr obesity intervention program, *CON* Control group, *EDU* Education, *ES* Effect size (regularly Cohen’s d if not specified otherwise), *EXP* Experimental group, LIFE* Multi-professional team, intensive wellness program (life-style, readiness, fitness, eating), MOVE!^§^ US Army weight loss program, *PA* Physical activity, *SAM* step activity monitor, ShipShape Navy weight loss program

^#^estimated from reported pre and post means, ≈ equal to

### Study quality (risk of bias)

The methodological quality was checked using the PEDro-Scale for randomized controlled trials (RCTs) and the NHLBI tool for observational or case-control studies according to the respective study design.

The non-randomized trials demonstrated a range of methodological quality from poor (three times 6–8 pts.), or moderate (eight times 9–10 pts.) to good (one time 11 pts.), meaning an acceptable quality status with no emerging risks of bias. The RCTs demonstrated PEDro scores ranging from 5 to 8 points indicating a fair (seven times 5–6 pts.) or good (twice 7–8 pts.) quality meaning an overall acceptable bias (Table 2).

### Intervention related effects on body weight or composition parameters

Over all, the literature review identified different approaches to improve body constitution parameters in military personnel. These ranged from physical activity monitoring programs [13, 14] to dietary programs alone [15] or in combination with physical activity [16], to educational or coaching programs [17, 18], to other different combined programs or pharmaceutical treatments including investigations with nutritional supplements [18, 19]. Some consecutive life-style modification studies were applied in the German Armed Forces [20–23] (Table 2).

#### Physical activity monitoring programs

##### Exercise only

Cederberg et al. examined the effects of an intensive and progressive endurance and strength training intervention over 6 to 12 months in a large scaled sample of Finnish military personnel (*n* = 1112, BMI ranging from normal to obese) [13]. They reported a high significant effect on body composition parameters as well as other cardiovascular disease (CVD) risk factors, without reporting any effect sizes or absolute post-test measures. They highlighted exercise associated fitness improvements to be correlated to CVD risk improvements. Weight changes of in average − 0.5 kg (± 5.2) or BMI changes of −0.3 kg/m² (± 1.7) or waist circumference changes of + 0.1 cm (± 5.5), and moreover fat mass or body fat percentage reductions of −1.3 kg (± 4.7) or −1,1% (± 4.6), respectively, with standard deviations not underpinning general and systematic positive exercise effects on body composition after 6 to 12 months intensive exercising in terms of absolute improvements.

In the framework of an initial fitness and conditioning program for officer candidates (Greek naval cadets, young and healthy, normal-weight BMI 23.3 kg/m²), intensive exercising (5 sessions per week: circuit strength and aerobic conditioning including team sports, swimming and obstacle running) for 10 weeks led to significant fitness improvements on the one hand and positive body composition changes (body mass – 1.8 kg, BMI−0.6 kg/m², body fat percentage −1,4%) on the other hand [13]. The corresponding moderate to large effect sizes of either ES = 1.29, or ES = 1.30, and ES = 0.60, respectively, were calculated retrospectively from the reported t-values [11]. However, these effects were observed in a normal weight population of military freshmen and should not be confused with the effects of body composition training effects in overweight or obese military personnel.

#### Dietary programs

##### Diet (low-fat or low-carb)

In their comparative study, Zinn et al. demonstrated that a twelve-week dietary intervention (low-carb vs. low-fat) was effective to reduce waist circumference and body weight. Low-carb led to a weight loss of −5.5 kg in average, and a reduced waist circumference of −4.8 cm, while a low-fat diet led to a decrease of −2.0 kg and − 3.3 cm. Thus, dietary effects within the investigated New Zealand military personnel were in favor of the low-carb diet in terms of body weight (−3.6 kg) and the waist circumference (−1.5 cm) with small effect sizes being likely clinically beneficial for weight loss (ES = 0.39) and probably beneficial for waist circumference reduction (ES = 0.21) [15].

##### Diet in form of supplements

Parastouei et al. reported the effects of a double-blinded, placebo-controlled trial using a synbiotic nutritional supplement (defined as combined pro- and pre-biotic microbial influencing lactobacillus mix) on body composition parameters, beside others, in a sample of military personnel suffering from a metabolic syndrome (*n* = 30 per group, BMI about 32 kg/m²) [24]. The authors reported age-adjusted large effect sizes (Cohen’s d > 0.8) between groups (verum vs. placebo) after the 8 weeks intervention on the BMI. In fact, the BMI and the waist circumference showed reductions of in average − 0.65 kg/m² (BMI) and − 2.64 cm (waist circumference) in the experimental group. These data were not directly reported by the original source authors. Thus, this had to be calculated by the authors of this review based on pre- and post-mean values. This resulted in the inability to calculate effect sizes within groups due to the lack of data.

##### Diet in the sense of meal-replacement

In the framework of the US Army ‘Weigh to Stay’ education-based program, Smith et al. evaluated probable favorable effects of a meal-replacement (Slim-Fast, 2 meals per day, 6 months maximally or the achievement of pre-set body composition goals) [17]. The participants in the “Weigh to Stay” program lost − 2.7 (± 4.4) kg (BMI: −0.1 ± 3.6). The participants who additionally replaced meals lost − 3.8 (± 3.5) kg (BMI: −1.1 ± 4.8). Fat mass was reduced by −2.7 (± 3.2) kg or −2.9 (± 2.5) kg, respectively. There were no statistically significant differences between ‘Weigh to Stay’ only and the additional meal-replacement groups. Effect sizes were not reported, but reconstructed They showed to be small (ES = 0.21 and 0.24) for weight loss, and moderate (ES = 0.66 and 0.50) for fat mass reduction [11].

#### Combination of dietary programs and physical activity monitoring

##### Physical activity (PA) promotion using wearable technology

In their mixed-methods study, Gorny et al. investigated the use and barriers of a mHealth approach (wearable technologies, wrist-worn step and heart rate tracker). The study investigated the promotion of physical activity during a 21-weeks-follow-up of the National Step Challenge (NSC) observation after a preceding 5-months weight-loss intervention for military personnel (Singapore Armed Forces, males, BMI > 27 kg/m²) [25]. The preceding weight-loss was about − 15.6 (± 4.1) kg. Weight regains at the end of the follow-up NSC intervention compared to the end of the weight-loss program was about + 5.3 (± 4.5) kg. This was accompanied by a decrease in tracker use from immediately after the first week (47%) to the twenty-first week (17%). Sustained users showed a slightly lower regain (+ 4.9 kg) than short-term users (+ 5.7 kg). PA (initially 10,576 steps per day) did not change significantly during the NSC observation period. Thus, the wearable tracker use showed not to be effective to prevent weight regain. Effect sizes were not reported and could not be calculated retrospectively.

##### Diet and exercise

LaFountain et al. investigated effects of a 12 weeks combined exercise and dietary intervention in a small sample of 15 (Exercise) and 14 (Control) overweight but in the majority not obese US military personnel (BMI 27.9 ± 2.9 kg/m²). They revealed positive body composition changes in favor of an ad libidum ketogenic diet in comparison to an unchanged mixed diet. The ketogenic diet achieved a weight loss of −7.7 kg (−3.5 to −13.6 kg), a body fat percentage reduction of −5.1% (−0.5 to −9.6%), a fat-mass reduction of −5.9 kg, and a loss of visceral fat-mass (−561.3 cm³) meaning a 43.7% visceral fat loss (3.0 to −66.3%), while the mixed diet remained almost unchanged in their body composition. Effect sizes were not reported by the authors and could not be calculated retrospectively from pre-post changes due to a lack of data reporting. The changes in body composition were meaningful high. Physical performance achievements after the exercise program were not significantly differently between both groups [16].

##### Short-term lifestyle intervention

In a sample of US Air Force members who failed to meet the US Air Force fitness assessment (211 males, 65 females), a single 4-hours lifestyle intervention (“Be Well” Course, including healthy nutrition and exercise benefits topics) was evaluated by Webber et al. 2021 with respect to fitness and body composition outcomes [26]. Along with significant fitness test improvements, BMI and waist circumference decreases were observed in males (BMI − 0.20 ± 1.02 kg/m² ES = 0.05, waist − 0.3 ± 1.23 inches equal to −0.76 ± 3.12 cm ES = 0.09) and in females (BMI − 0.20 ± 0.87 kg/m² ES = 0.06, waist − 0.85 ± 1.67 inches equal to −2.16 ± 4.24 cm ES = 0.04), respectively. Effect sizes were calculated retrospectively leading to Cohen’s d values of smaller than 0.1, meaning a trivial effect size, only [11].

#### Educational and coaching programs

##### Education booklet and weekly emails for weight gain prevention/weight loss

Targeting annual weight-gain prevention (educational booklet with information and weekly motivation and compliance emails including physical activity and dietary recommendations) was evaluated without in-person contact in a large-scaled (*n* = 3,502) and controlled trial (*n* = 65,089) sample of US Air Force members showing a pre-overweight and pre-obese status (baseline age about 31 years, about 12% females, BMI from 24.0 to 29.9 kg/m²) [14]. Robbins et al. reported differences for subgroup-analyses with a small weight loss for women (equal to −0.01 kg compared to + 0.36 kg within controls), and with a comparably small weight loss (equal to −0.32 kg compared to + 0.27 kg within controls) for Senior Airmen (pay grade E-4), but with weight gains in all other males (equal to + 1.3 kg compared to + 1.1 kg within controls) after 12 months. Standard deviations, any other confidence intervals or effect sizes were not reported, and could not be calculated retrospectively due to lacking data.

##### Education internet-based for weight gain prevention/weight loss

Targeting weight-gain prevention, beside weight-loss, was evaluated over a 6-months RCT using an internet-based behavioral treatment (including motivation calls after 4 and 8 weeks) compared to usual care [27]. The authors emphasize that weight-gain prevention was successful, although 42% of participants demonstrated weight-gains (60% within the controls). For the experimental group, weight loss was − 1.3 kg (± 4.1), BMI decreased by −0.5 kg/m² (± 1.4), percent body fat deceased by −0.4% (± 3.1), and waist circumference showed a reduction of −2.1 cm (± 4.3), which was rated as a small effect in favor of the internet-based program. Statistical effect sizes were not reported and could also not be calculated retrospectively.

##### Education internet-based for weight loss with or without using promotion program

Stewart et al. conducted an internet-based weight loss education program (including ‘food planer’, ‘exercise planer’, and ‘lifestyle planer’ components) with or without an additional tool promoting the web-site use and found that the study goal – achieving a self-reported weight loss of at least 5% compared to baseline weight – was met better the more frequently the use of the promotion tool was (*r* = −0.21, *p* < 0.0001) [28]. In average, 12% of the total of the participants achieved a 5% weight loss at any time during the intervention. Unfortunately, baseline weight or weight after the end of the intervention or any effect sizes were not reported, and could not be calculated either due to lacking data.

##### Preferred type of daily diary/adherence and BC parameters

Using one’s preferred type of daily diary (paper, PDA, web-based) influences the use of a daily diary in favor of using the preferred method (food-intake: 62.2% vs. 43.4% and exercising: 60.6% vs. 31.2%) in a sample of active duty military members being at least overweight but in average obese (BMI ≥ 25 kg/m², baseline BMI 33 ± 3.5 kg/m²) [29]. But changes of body weight or composition did not differ between these comparison groups after 12 weeks showing decreases in weight (−2.7 ± 3.9 kg), body fat percentage (−1,6 ± 2.6%), and waist circumference (−2.2 ± 2.2 inch equal to −5.6 ± 5.6 cm) referring to the total sample (*n* = 39, 95% males). Effect sizes were not reported and could not be calculated retrospectively.

#### Other combined programs

##### Coaching (plus herbal supplement)

McCarthy et al. explored additional body composition modification effects of a specific controlled coaching program (Nurse Health Coaching) with or without a placebo-controlled herbal supplement application after a completed weight loss US Army program (MOVE!) [18]. The authors determined statistical effect sizes for body composition changes in favor of the Nursing program groups compared to the waiting control group. At the end of this 12-week intervention, effect sizes in favor of Nurse Coaching ranged from 0.07 to 0.11 (Cohen’s d) with absolute values for Coaching group improvements of in average − 2,0 kg, for weight loss, −0.65 kg/m² for BMI and − 1.3 cm for waist circumference, as well as −1.0% for body fat percentage, respectively.

##### PA promotion/education

Sanaeinasab et al. observed significantly better outcomes for their experimental group. It was compared to the controls after 7 educational sessions promoting weight management due to increasing general physical activity (PA) [30]. Self-reported PA increased by about + 57% (ES = 0.57) and BMI (ES = 0.54) decreased by about − 3.5% after 12 weeks. This means a moderate effect size for their overweight to obese sample (BMI 29.0 ± 3.5 kg/m²).

##### Complex life-style-change-program

As a combination of cognitive-behavioral, psychoeducational, exercise, and nutritional principles, a life-style wellness program (outpatient, group treatment) was evaluated among US military personnel typically demonstrating an overweight or obese body constitution status (BMI about 30.5 kg/m²) [31]. The program began with an intense week (phase 1: individually tailored programs including exercising, nutritional education and life-style behavior leading to a better self-awareness and decision-making cycles) followed by a 1-year follow-up (phase 2: ongoing group-sessions). The life-style program resulted in significant weight losses of about 11 and 14 lbs (equal to −4.9 kg and − 6.4 kg) for males and females, respectively and accordingly to BMI reductions of about − 2 kg/m² predominantly during the first 6 months of the observation. Effect sizes (Cohen’s d) for these improvements were calculated retrospectively and ranged from 1.18 to 1.39, meaning large effects [11].

Another intensive lifestyle intervention program (Fit Blue) aimed to translate the US Army ‘Look AHEAD’ weight loss program under conditions of distance-based counselor-initiated or self-paced program conditions [32]. The complex lifestyle intervention consisted of individualized and progressive exercise prescription videos, educational and motivational sessions, daily weight monitoring displayed in a web-based tool, meal-replacement offers or dietary meal plans including a food scale showed better outcomes for the counselor initiated than for the self-paced option after 4 months (weight loss phase: −3.2 ± 3.4 kg and 29.8% participants with ≥ 5% weight loss vs. −0.6 ± 2.9 kg and 10.5% participants with ≥ 5% weight loss) and 12 months (sustainability of weight loss: −1.9 ± 4.1 kg and 29.5% participants with ≥ 5% weight loss vs. −0.1 ± 3.8 kg and 15.6% participants with ≥ 5% weight loss) in overweight (43.5%) or obese (56.5%) military personnel. Effect sizes were not reported and retrospective calculations were not applicable.

Among obese German Army soldiers (n = 1090, BMI 33.8 ± 4.0) a complex but somehow more simply structured weight loss program including individualized endurance exercise prescriptions and health risk factor awareness with dietary counseling suffered from an enormous drop-out rate (about 60%), and led to weight losses of in average 7 kg (out-patient study arm) or 4.4 kg (course-based study arm) [21]. Effect sizes were not reported and could not be calculated retrospectively, but the authors concluded that program goals of 5% or 10% body weight loss rates were not achieved. Results of the out-patient study arm (n = 665) were reported separately (after one year: body weight − 3.1 kg, BMI − 0.9 kg/m², waist circumference − 3.1 cm) without effect sizes but highlighting that 12.2% and 8.4% of participants achieved a 5% body or 10% weight loss, respectively, and the course-based study results arm were published separately, again [22]. Drop-out was described as 46.1% and only a small number of 27.5% of the completing participants showed a weight loss of ≥ 5% body weight with statistically significant but absolutely too small effects of about − 4 kg weight loss and − 4 cm waist circumference [23].

##### Pharmacological treatment plus fat-reduction-diet

For the uptake of fat energy absorption reducing medication (3 pills à 60 mg Orlistat per day over 6 months) while maintaining a fat-reduction-diet Smith et al. found placebo-controlled effects in a clinical trial including obese US Army soldiers (BMI > 30 kg/m²) with trivial to small effect sizes (Cohen’s d from 0.02 to 0.37) [19]. Orlistat led to a body weight reduction of −3.1 (± 4.7) kg (ES = 0.19), to a fat mass reduction of −2.5 (± 3.9) kg (ES = 0.37), to a fat-free mass reduction of −0.3 (± 2.8) kg (ES = 0.02), and a BMI reduction of −1.1 (± 1.7) kg/m² (ES = 0.33) in a per-protocol-analysis. The effect sizes were calculated retrospectively [11]. Placebo – representing in fact a fat-reduction-diet condition – led to a comparable weight loss and BMI change, but a smaller fat mass and greater fat-free mass loss.

## Discussion

This review aimed to identify weight management approaches in military personnel environments targeting weight-loss and weight-gain prevention as countermeasures against widespread overweight and obesity among members of the Armed Forces potentially hampering their military readiness and physical health state. In order to compare the effectiveness, literature findings shall be discussed beginning with exercise only interventions, diet only interventions and combined interventions. The order of the discussed studies is according to their ranking of reported or retrospectively calculated statistical effect sizes (ES) for body composition improvements from smaller to larger ES.

The two studies with exercise only interventions were described by Cederberg et al. [13] and Vantarakis et al. [33].Almost negligible body composition improvements were found for a progressive endurance and strength training covering a six to twelve months intervention in normal weight to obese military personnel [Cederberg et al., 2011] [13]. These exercise only induced effects of weight (−0.5 kg), BMI (−0.3 kg/m²), waist circumference (−0.1 cm), and body fat percentage (−1.1%) reductions were reported without statistical ES, but decreases remained far from those described for endurance training induced effects in the general population, as reported in a meta-analysis for example for waist circumference reductions (−3.2 cm (95% −3.6 to −2.6)) [34]. Exercise only effects on weight loss were described in an earlier meta-analysis as being about 2.9 (± 0.4) kg in short-term interventions in the general population, which was even more than in military personnel environments [13, 35]. In contrast, the above-mentioned body fat percentage changes (−1.1%) were almost comparable to resistance training only effects (−1.6%), as reported for the general population in another meta-analysis, while a combined strength and endurance training led to larger body fat percentage decreases (−2.3%), and in combination with caloric restriction, body fat percentage decreases (−3.8%) were even more pronounced [36].

Moderate to large ES (0.6 − 1.3) were retrospectively calculated for an intensive exercise program leading to meaningful fat mass percentage decreases (1,6%) and weight-losses (1.8 kg), but these effects referred to a normal-weight sample of cadets at the beginning of their military service – meaning at the transition from civilian to military in-duty lifestyle conditions at the Greek Navy – with the primary target to improve their physical fitness [33]. As such, these achievements were not really good comparable to study results dealing with obese military personnel seeking for weight reductions.

Two dietary interventions were described by Zinn et al. [15] and Parastouei et al. [24].

Dietary approaches were somewhat more promising. A low-carb diet achieved a favorable weight-loss (−5.5 kg) and waist circumference reduction (−4.8 cm) compared to a low-fat diet (−2.0 kg and 3.3 cm) [15]. Both, low-carb and low-fat diets achieved better benefits than any other above-mentioned intervention. Although statistical ES were not reported for the separate diet variants, low-carb weight reductions were somewhat in the range of short-term results (−12.2 to −0.3 kg) reported in a review article dealing with the general obese population either with or without diabetes mellitus type II or even another meta-analysis describing BMI reductions of about − 2 kg/m² (6%) after dietary counseling in the general population [37, 38]. The favorable low-carb effect on body weight was described as being likely clinically beneficial compared to low-fat; the low-carb effect on waist circumference was described as being probably clinically beneficial compared to low-fat as highlighted by the authors [15].

Meal-replacement strategies (twice a day formula diet, Slim-Fast) did not reveal additional advantages in weight loss or fat mass reduction compared to an education-based program alone (Weigh to Stay, US Army), although the authors reported effects in favor of an additional meal-replacement, if an intent-to-treat analysis was applied [Smith et al., 2010] [17]. But retrospectively calculated effect sizes were only small for widely varying weight-losses of about three to four kilo grams, or probably moderate for fat mass reductions of about three kilo grams after six months either varying widely. BMI reductions associated with formula diet usage of about one kilo gram per square meter were slightly higher than due to the application of synbiotic (microbiome) supplements [24].

The following studies described combined interventions in the order of ascending effect sizes (trivial < 0.2, small ≥ 0.2, moderate ≥ 0.5 and large ≥ 0.8).Only trivial ES were assessed for a single (4 h) educational lifestyle changing approach in Air Force members not meeting fitness recommendations by Webber at al. in 2012, although this punctual short-term intervention was reported to be effective as a countermeasure by the authors [26]. Actually, ES were modest (< 0.10) for BMI reductions of about 0.2 kilo grams or waist circumference reductions for males and females of in average 0.8 cm and 2.2 cm, respectively, after a period of two to three months (interquartile range).

Low ES were also determined for additional body composition improvements in favor of a Nurse Health Coaching program compared to controls, which were documented after a 12-week program after a preceding US Army weight loss standard program (MOVE!-study) [18].

Despite ES were not reported and the study goal focused more on weight gain prevention than on body composition improvements, another educational approach should be discussed as probably not being favorable. Robbins et al. claimed for their booklet and motivation email-based intervention also a successful weight gain prevention effect, because a normally expected annual weight gain of about a half to one kilogram (+ 0.45 to 0.9 kg) was not observed in their total sample [14]. In this study, weight losses were achieved minimally only in women (−0.01 kg) and in a minority of male Senior Airmen (−0.32 kg), but the majority of other male Air Force members gained body weight (+ 1.3 kg) after twelve months, exceeding the regularly expected weight gain of less than one kilo grams. Thus, the effectiveness of educational and motivation promoting approach may remain a matter of debate.

Somehow similar, internet-based behavioral education effects targeting weight gain preventions appear to be ambiguous, although the authors concluded that their approach to promote weight-gain prevention was successful due to a lower rate of 42% weight-gainers compared to 60% within their controls [27]. ES were also not reported and could not be assessed retrospectively, but weight-loss effects after six months were rather small or even negligible and individually varying widely (weight − 1.3 ± 4.1 kg, BMI − 0.5 ± 1.4 kg/m², BF% −0.4 ± 3.1%). Thus, the authors’ interpretations of success may also remain a matter of debate.

Although ES were not reported and not retrospectively applicable, the use of wearable technologies (fitness trackers) to promote physical activity after a successful weight loss program showed to be not effective to prevent weight regain, which actually was about five kilo grams after 21 weeks follow-up, although the higher adherence to use the trackers was accompanied by slightly smaller regains [25].

Somehow as an add-on to web-based approaches, Stewart et al. found that an additional tool to promote the use of internet-based educational programs including ‘food planer’, ‘exercise planer’, and ‘lifestyle planer’ components led to better weight loss results [28]. But neither baseline nor follow-up body weight measures were reported, except the success rate of about 12% of the participants having achieved a 5% body weight loss. Accordingly, it was not easily possible to rate the effectiveness of this weight loss program approach in absolute numbers or even effect sizes. Probably, a comparison with a double-blinded placebo-controlled clinical trial among the general, overweight or obese population allows an interpretation [8]. Wilding et al. found weight loss rates (≥ 5% body weight reduction) in their controls (BMI ≥ 30 kg/m², lifestyle intervention program beside a placebo-pharmaceutical treatment) of 31.5%, and a body weight loss of 10% or more in 12% of these participants. This would enable us to resume the above-mentioned lifestyle program effect of 12% of the participants achieving a 5% body weight loss as being markedly smaller compared to 12% of lifestyle-program participants (study population controls) achieving a 10% body weight loss [8, 28].

Using a daily diary for food-uptake and exercising may be a more favorable approach to achieve a systematic weight loss, as reported by Shay et al., while the type of a used diary did affect the adherence, but not the amount of weight loss or body fat percentage and waist circumference decreases [29]. Despite larger positive body composition changes – compared to any above-mentioned approaches – the individuals’ changes varied markedly, and the authors themselves concluded that body composition changes did not reach a clinically meaningful level.

In comparison to military personnel, larger effects for dietary or diet and exercise combinations were observed in the general population. Diet only weight loss effects (−10.7 ± 0.5 kg) showed to be much larger than exercise only effects (−2.9 ± 0.4 kg), but the combination of diet and exercise was even a little better (−11.0 ± 0.6 kg) in short-term (< 6 months) interventions, although these effects shrank a little over time (−8.6 ± 0.8 kg) [35].

Larger effects were observed for pharmaceutical approaches. Facing the effectiveness for body weight decreases induced by pharmaceutical products reducing the fat-energy uptake (Orlistat), weight loss in an obese sample of military personnel of about three kilograms was effective but a little less pronounced than in the large scaled obese sample in the general population, which was reported as being about five and a half kilograms after a six months medication period based on meta-analytical data [19, 39]. But Wilding et al. reports compared to the body weight reduction effects of about fifteen kilo grams after subcutaneous injections of Semaglutide (2.4 mg/week) – a glucagon-like peptid-1 receptor-agonist, oral fat-absorption reducing pharmaceutical effects appear to be minor but still meaningful [8].

At least moderate ES of larger than 0.5 were assessed for a promotion of physical activity (increases of more than 50%) accompanied by BMI improvements (3.5%) in an obese sample of military personnel after a 12 weeks educational program, which showed to have larger ES than reported for short-term behavioral changing techniques promoting either physical activity and healthy eating (ES 0.37 CI 0,26–0.48) in a meta-regression analysis for the general population [30, 40].

Without reported statistical ES, but demonstrating the greatest values for body composition improvements, an ad libitum ketogenic diet combined with exercising revealed an average weight loss of almost eight kilo grams accompanied by percent body fat reductions of in average 5% in overweight but not obese military personnel [16]. These results were comparable to those identified in a review article covering ketogenic diets in the general population describing even larger weight losses of in average almost nine kilo grams [41].

The largest ES (> 1.0) for observed weight-losses predominantly during the first six months of the observation period (BMI − 2 kg/m² for both sexes, weight loss males − 4.9 kg and females − 6.4 kg) were calculated retrospectively by Bowles et al. for a complex lifestyle changing program consisting of a combination of individually tailored cognitive-behavioral, psychoeducational, exercise, and nutritional principles with ongoing sessions for a follow-up of one year, which was leading to a better self-awareness and decision-making cycles [31]. A weight loss of 7.7 kg in ketogenic diets is clinically meaningful but limited by small samples.

Also, a complex lifestyle changing program, but a little less effective for clinical weight loss after a four months weight loss (−3.2 kg) or a twelve months sustainability phase (−1.9 kg) compared to Bowles et al. was reported by Krukowski et al., unfortunately without reporting statistical ES [32]. But the results of distance-based and counselor-initiated interventions were described as a promising approach for obese US military personnel. For the general population, Baillot et al. conducted a meta-analysis and found shot-term (up to six months) and prolonged (more than six months) weight loss effects of either − 7.2 [CI −8.88 to −5.52] kg and of −11.33 [CI −13.07 to −9.59] kg [42]. This underpinned that comprehensive complex combinations of lifestyle changes in combination with exercise promotion and dietary measures were the most effective interventions over the short or a prolonged time frame compared to other approaches, although those applications in the military environment were less effective compared to the general population, which was apparent especially in a longer time frame, where general population results demonstrated larger weight losses, while military personnel showed a weight regain apparent as smaller weight losses [21–23, 31, 32].

Applications of complex individualized exercise prescription., health-risk associated lifestyle modifications and nutrition habits counseling among the German Armed Forces (Bundeswehr, only one data set reported in three consecutive publications) did not achieve self-set program goals of at least 50% of participants demonstrating a 5% or 10% body-weight loss after 18 to 24 months [21]. Results for a subsample (out-patient study arm) of these data published originally in German language were published separately either without giving ES. Weight losses within the remaining sample after high drop-out rates of about 3.1 kg and waist circumference reductions of about 3.1 cm after one year were deemed to be no more than moderate [22]. And the other study arm data (course-based) were published separately either – again emphasizing the high drop-out rate of almost 50% with only 27% achieving a body weight loss of at least 5% meaning that the program was less successful than expected, These results did not meet expectations due to high dropout rates and modest weight loss [23]. In order to improve the military fitness and operational readiness of military personnel, combined intervention programs should be integrated into service operations. Annual fitness training in conjunction with health checks before and after deployment could potentially provide promising approaches here. Stress in the workplace and readiness duties reduce the opportunities for adequate physical activity during the diverse professional obligations of routine operations. Official guidelines for interventions through policy recommendations, such as regular wellness activities for soldiers with elevated BMI, could bring about possible improvements in this area. To evaluate the effect of pharmaceutical interventions future RCTs should evaluate for example Semaglutide in military populations.

### Limitations

There are some limitations in our study. One limitation that complicates the interpretation of our results relates to the statistical effect size, which was not consistently reported by the authors of the original sources and in some cases could not be calculated retrospectively. This was hampering our objective to determine a ranking order of a suggested effectiveness of overweight and obesity countermeasures in military personnel environments. In those cases, we considered the absolute mean changes of reported body constitution parameters as an alternative option for the intended ranking of countermeasures, but in these cases, we had to accept a potential bias due to statistical basic requirements. Another limitation is the start of a preliminary literature search on July 16.2024. After refining the study protocol, we started the registration in PROSPERO on December 15, 2024. Further limitations are a potential publication bias, as predominantly positive findings may suggest bias (e.g. funnel plot analysis was not feasible). Due to hand search some studies were found that have no doi or internet source (e.g. Brandes et al.) [20]. The absence of control groups in some studies reduce the quality of significance. Some studies reported about a high dropout rate up to 60%, like Sammito et al. [21, 22] and the connected impact and a reduced generalizability. There are additionally some specific barriers to meta-analysis (e.g. varying outcomes like BMI vs. waist circumference). Standard deviation was not specified in all studies used. Different dropout rates in the available studies could also influence the estimated ES, as only those study participants who successfully completed the study were taken into account. Overall, there is little ES data available from the studies.

## Conclusions

The present results show that combined lifestyle interventions involving behavioral changes, physical activity, and nutrition-based interventions have the greatest effect size in military personnel compared to single interventions. Combined interventions appear promising but require further validation due to study heterogeneity. Single interventions involving exclusively dietary measures, nutritional replacement products (formula diets), exercise interventions, and behavioral interventions showed lower effect sizes in terms of their impact on weight loss in overweight and, in particular, obese military personnel. Regardless of weight reduction, the effect of interventions on physical fitness should not be ignored, which was not specified in these studies, as the specific nature of military work requires a high level of readiness. High-quality RCTs are needed to confirm efficacy in military settings and in addition, studies should be conducted on long-term sustainability or interventions tailored to military roles. Future research could likely include the promotion of training and physical activity in combination with new pharmaceutical approaches (e.g., semaglutide or bimagrumab).

## Supplementary information

Supplementary Material 1. Appendix A Study table with list of all included studies

## Data Availability

No datasets were generated or analysed during the current study.

## Abbreviations

BMI: Body mass index
CCT: Clinical controlled trial
CI: Confidence interval
CVD: Cardiovascular disease
ES: Effect size
HIIT: High intensity interval training
mHealth: Mobile Health
n: Number
NHLBI: National Heart Lung and Blood Institute
RCT: Randomized controlled trial
