# Supplementary material for: Prevention and intervention against obesity and overweight in the military: a systematic review
Source: J Occup Med Toxicol. 2025 Oct 7;20:32. doi: 10.1186/s12995-025-00480-7 (PMC12502539; doi:10.1186/s12995-025-00480-7)
Supplement: Supplementary file 1 — Supplementary Material 1. Appendix A Study table with list of all included studies [file 12995_2025_480_MOESM1_ESM.docx]

**Appendix A**

Study table with list of included studies

| Study Details | Population | Counter Measure | Outcome | Study Quality |
| --- | --- | --- | --- | --- |
| Author: Zinn et al  Year: 2017  Region: New Zeeland  Title: A 12-week low-carbohydrate, high-fat diet improves metabolic health outcomes over a control diet in a randomised controlled trial with overweight defence force personnel[^1^].  Study Type: RCT | **overall**: n= 41 **male:** n= 27 **female:** n= 14  **Baseline Characteristics:**  **LCHF:** n=21,  male: 16 (76%), female: 5 (24%),  Age: 39.6±7.8 years  BMI: Female 31.2±5.5, Male 31.7±4.6;  Waist (cm): Female 85.6±14.8 Male 101.3±15.1  **Control:** n=20,  male: 11 (55%), female: 9 (45%),  Age: 39.7±9.6 years,  BMI: Female 30.8±3.8kg/m², Male 30.5±3.0 kg/m² Waist: Female 92.2±12.6 Male 101.1±13.9  **Exclusion criteria:**  Participants who consume 250g of carbohydrates or less in everyday life; BMI <25kg/m² | **Group:** Diet  12-week LCHF (low carbohydrate high fat) vs control diet: high carbohydrate, low fat | **LCHF (n=14):**  Baseline / Week 12  Weight (kg) 96.2±13.3 / 90.7±11.8, p<0.05;  Waist (cm) 98.0±11.9/ 93.2±10.3, p<0.05,  significant: HDLc (mmol/l)↑, TG (mmol/l) ↓, GCG (mmol/l)↓; self-reported adherence: (mean ± sd) 77.0 ± 19.9% p<0.05, FFQ dietary: 18.0 ± 7.4;   **Control (n=12)**:  Baseline/12-week;  Weight (kg) 94.8±11.8/92.8±12.2p<0.05;  Waist (cm) 100.8±10.5/97.5±10.2, p<0.05,  self-reported adherence (mean ± sd) 59.1 ± 20.6% p<0.05,  FFQ dietary: 22.7 ± 11.7   Between group Cohens d )´(90% CI): Weight: −0.39 (−0.64, −0.14) - Effect size small Waist: -0.21 (−0.45, 0.04) -Effect size small  37% **attrition rate** | PEDro 5/11 |
| Author: Robbins et al  Year: 2006  Region: USA  Title: A low-intensity intervention to prevent annual weight gain in active duty Air Force members [^2^].  Study Type: CCT | **overall:** n= 68,591 **male:** n= 59,820 **female:** n=8,771  **Baseline characteristics:** **Intervention**  n=3,502; age (mean): 31.9,  male: 87%, female: 13%  **Control**  n=65,089;  age (mean): 30.4,  male: 90%, female: 11%  **Inclusion criteria:** BMI at baseline ranged from 24.0 to 29.9 kg/m2  **Exclusion criteria:**  BMI >30 -> USAF body fat program | **Group:** Life Style intervention  two PEP booklets and 52 weekly e-mails, Low-Intensity Intervention | intervention was completely effective at preventing weight gain  **mean weight change (lbs)**  men (1) n=7,518:  Intervention -0,7  Control +0,6  difference -1.3 p=0.016  men (2) n=52,302:  Intervention +2.8  control +2.5  difference +0.3 p>0.05 (not significant)  women n=8,771:  Intervention -0.2  control +0.8  difference -1.0 p=0.031 | NHLBI 9/14 |
| Author: McCarthy et al  Year: 2017  Region: USA  Title: A Randomized Controlled Trial of Nurse Coaching vs. Herbal Supplementation for Weight Reduction in Soldiers [^3^].  Study Type: RCT | **overall:** n= 435 **male**: n= 319 (73.4%) **female:** n=116 (26.6%)  **Baseline Characteristics:** mean age: 30 ± 8.2,   **Randomized study group:**  n=335 control n=86, coach n=81, supplement n=83, placebo n=85 Weight (lbs): Control: 217.08 (33.32) Coach: 217.27 (38.86) Supplement: 218.87 (30.54) Placebo:214.12 (30.25) BMI: Control: 32.45 (3.19) Coach: 32.44 (3.65) Supplement: 32.25 (3.2) Placebo: 32.63 (3.02) WC (in): Control: 38.53 (3.87) Coach: 38.22 (4.16) Supplement: 38.65 (3.4) Placebo: 38.13 (3.65)  **Self-referred group: n=100** Weight (lbs): 206.79 (38.48) BMI: 30.53 (3.78) WC (in): 37.01 (4.41)  **Inclusion criteria**: Age > 18y, >3 months remaining service time, not a participant in the MOVE! And generally healthy according to self-report  **Exclusion criteria:**  women until 6 months postpartum or breastfeeding, anyone with an endocrine abnormality, eating disorder, or taking medications contraindicated with Garcinia Cambogia (G. cambogia) | **Group:** Coaching & Life Style & diet (suppl.)  12-week-intervention nurse coaching with and without an herbal supplement vs herbal supplementation (G.cambogia)  fifth arm: self-referred (7 sessions to healthy living and behavior change to achieve weight and fitness goals | documented at baseline, 6 weeks, and 12 weeks;  **Control vs. Combined Coaching Groups, Week 12 Change Scores** Weight (lbs) Control: −2.22 (0.56) Coach: −4.39 (0.66) Difference: −2.17 (0.86) Cohens d: −0.07 p: 0.012 BMI: Control: −0.32 (0.08) Coach: −0.65 (0.1) Difference: −0.33 (0.13) Cohens d: −0.10 p: 0.011 WC (in) Control: −0.31 (0.1) Coach: −0.56 (0.12) Difference: −0.25 (0.16) Cohens d: −0.06 p:0.118   **adherence score**:  Control: 8.92% (±4.19) self-referred group: 53.78% (±22.56), NHC 47.1%, NHC+supplement 40.7%, NHC+placebo 41.1% p<0.0001  **significant between self-referred and control:**  25(OH)D, HDL, Trig, HDL:CHOL,  Weight (lbs) CG: 217.08 (33.32) vs SR: 206.79 (38.48)p= 0.05 BMI CG: 32.45 (3.19) vs SR: 30.53 (3.78) p= 0.0003 | PEDro 6/11 |
| Author: Sanaeinasab et al  Year: 2020  Region: Iran  Title: A Theory of Planned Behavior-Based Program to Increase Physical Activity in Overweight/Obese Military Personnel: A Randomised Controlled Trial [^4^].  Study Type: RCT | **overall:** n= 84 **male**: n= 84 **female**: n= 0 Intervention: n=42, control: n=42  **Baseline Characteristics:** mean age: 40.4 (SD 7), average BMI: 29 (SD:3.5)  **Intervention group:**  Age <35 years: 11(26.2%) >35 years: 31 (73.8%), BMI: 25-30: 29 (69%), >30: 13 (31%)  **Control:**  Age <35 years: 9 (21.4%) >35 years: 33 (78.6%), BMI: 25-30: 24 (57.1%), >30: 18 (42.9%)  **Inclusion criteria:**  obese or overweight (BMI >25), employment for at least 2 years  **Exclusion criteria:**  relocation during next 6 months, history of chronic heart failure, severe kidney disease or other disabilities that might interfere with intervention program, involved in other weight management program, medication to reduce weight | **Group:** Life Style intervention  health education on behavior change  7 sessions | Data collection at baseline and 3 months later  **Measures at Follow-up in Intervention and control group:** BMI:  Intervention: 27.6 (2.7) Control: 29.1 (3.8)  within group (baseline to follow-up): Intervention: p<0.001 t=3.52, Control: p=117 t=1.21,  between group: p=0.040 t= -2.08, Effect size: 0.39  significantly improved physical activity in intervention group (not sign. in control group) | PEDro 6/11 |
| Author: Shay et al  Year: 2009  Region: USA  Title: Adherence and weight loss outcomes associated with food-exercise diary preference in a military weight management program [^5^].  Study Type: RCT | **overall:** n= 73 (39 after loss to follow up) **male:** n= 23 (59%) **female:** n= 16 (41%)  **Baseline Characteristics**:  Age: 35,33 (±9.3),  BMI: 33.0 (±3.4), Body fat: 34.9% (±7.3),  Waist circ.: 41 (±4.4) WEL-Score: 115.5 (±29..9)  **Inclusion criteria**: BMI >25kg/m², enrolled in 8-week- ShipShape Navy Weight Program  **Exclusion criteria:**  pregnant | **Group**: Life Style intervention  ShipShape: 8 sessions a 90min once per week. record daily energy balance on paper, PDA ord web-based diary. Testing if the preferred group works better, 12 week study, self-monitoring method | **Change in body composition and self-efficacy (WEL) scores from baseline to 6 weeks, 6 to 12 and baseline to 12:**  Weight kg: 6wks: -2.4 (±2.7) (p<0.25), 6 to 12wks: − 0.3 ± 2.2, 12wks: -2,7 (±3.9) Body fat (%): 6 wks: − 1.1 ± 1.9, 6 to 12 wks: − 0.5 ± 2.1, 12 wks: − 1.6 ± 2.6 Waist (in): 6wks: − 1.6 ± 1.7, 6 to 12 wks: − 0.6 ± 1.9, 12 wks: − 2.2 ± 2.2  **no significant interaction between diary preference and time for any of the three variables**  (F(2,36) = .10, p = .90 for weight, F(2,36) = .92,p= .40 for % BF, and F (2,36) = 1.8, p = .18 for waist circumference). **week 12 subjects as a whole demonstrated a significant decrease in** weight (−2.8 kg., p b .001), waist circumference (− 2.2 in., p b .001) and estimated percent body fat (− 1.6%, p b .001) **no significant difference between preferred and non-preferred** **no statistical difference between the three options** | PEDro 6/11 |
| Author: Gorny et al  Year: 2022  Region: Singapore  Title: Active Use and Engagement in an mHealth Initiative Among Young Men With Obesity: Mixed Methods Study[^6^].  Study Type: Mixed Method | **overall:** n= 167 **male:** n= 167 **female**: n= 0  **Baseline characteristics:** age: 21 to 25 years  119 not contacted contacted: 48 -> declined follow up: 19 (11%), attended follow-up and completed survey: 29 (18%)  **Exclusion criteria:**  Age <21 years | **Group:** Exercise  Step activity monitor (SAM) | questionnaires, interviews, tracker data  study participants n=29: Sustained: n=13, short-term n=16  **mean weight measures kg (SD):**  Upon entry into residential program: overall: 99.4 (11) sustained users; 97.4 (14) short-term-users 101 (8) p=0.40 Upon enrolment into NSC: overall: 83.8 (10.4), sust.: 81.9 (13.7), short:85.4 (6.6), p=0.38 At time of study: overall: 89.2 (10.2), sust.:86.8 (12.7), short: 91.1 (7.7), p=0.26  **mean BMI:** follow-up study: overall: 29.6 (3.1), Sust.: 29.3 (3.8), short: 30 (2.5), p=0.56 | NHLBI 8/14 |
| Author: Krukowski et al  Year: 2018  Region: USA  Title: Dissemination of the Look AHEAD Intensive Lifestyle Intervention in the United States Military: A Randomized Controlled Trial [^7^].  Study Type: RCT | **overall:** n= 248 **male:** n= 122 (49.2%) **female:** n= 126 (50.8%) CI Condition: n=124, SP condition n=124  **Baseline Characteristics**:  Age: 34.6 (±7. 5) BMI: Overweight: 108 (43,5%), obesity 140(56,5%).  Weight: 88kg (±14.3)  **Inclusion criteria:** active duty military personnel at Lackland AFB in San Antonio Texas,  at least 1 year left there,  >18y, BMI>25,  computer and email access, clearance from healthcare provider  **Exclusion criteria:**  medical conditions that would impact weight loss: uncontrolled hypertension, disability or condition that would limit regular aerobic exercise, a history of cerebral, coronary or peripheral vascular disease or uncontrolled cardiac arrhythmia; uncontrolled congestive heart failure in the past 12 months; a history of significant kidney or liver disease; presence of uncontrolled thyroid disease or pheochromocytoma; a malignancy (other than non-melanoma skin cancer) in the last 5 years; presence of diabetes mellitus treated with a medication that could cause hypoglycaemia; presence of unstable emotional or psychiatric condition; current use of medication that influences weight; current pregnancy, a child birth within the last 6 months, or planning to become pregnant during the study period; and a history of bariatric surgery or significant recent weight loss (i.e., greater than 10 pounds in the past 3 months). Potential participants must not have had more than one failure of the military-proctored physical fitness test in the past 12 months, | **Group:** Life Style intervention  Fit Blue Study  Look Ahead Intensive lifestyle intervention, 1:1 ratio to the Counsellor-Initiated intervention (1) and the Self-Paced intervention (2), 1: 28 one on one telefon for 12 months with trainer in behavior change skills | 4 months visit: n=199 (CI 109, SP 90) 12 month visit: n=172 (CI 95, SP 77)  **weight loss 4months:**  CI, mean ± SD = −3.2 ± 3.4 kg vs. SP, −0.6± 2.9 kg; P< 0.0001;  percent weight loss: CI, 3.5% ± 3.8% vs. SP, 0.6% ± 3.1%; P < 0.0001 participants who lost 5% or more: CI: 29.8%; SP: 10.5%; P < 0.001 mean abdominal circumference reduction: CI: 3.5 ± 6.0 cm , SP: 1.2± 4.1 cm , p<0.0001  **weight loss 12 months:** CI: mean ± SD = −1.9 ± 4.1 kg; SP: −0.1 ± 3.8 kg; P < 0.001 percent weight loss: CI: mean ± SD = 2.1%± 4.7%; SP: 0.0% ± 4.0%; P < 0.001 lost 5% or more at 12 months (CI: 29.5%; SP: 15.6%; P < 0.05 Mean abdominal circumference: CI: 2.7± 6.5 cm, SP: 1.7 ± 8.1 cm (P < 0.05) | PEDro 5/11 |
| Author: Smith et al  Year: 2010  Region: USA  Title: Efficacy of a Meal-Replacement Program for Promoting Blood Lipid Changes and Weight and Body Fat Loss in US Army Soldiers[^8^].  Study Type: RCT | **overall:** n= 113 **male:** n= 76 **female:** n= 37  weight to stay: n=56, meal replacer: 57  **Baseline Characteristics:**  Weight (kg): Weigh to Stay: 56 99.1 ±14.1, meal replacers: 98.0±16.4 BMI: Weigh to Stay: 33.1±2.9, meal replacers: 33.1±3  **Inclusion criteria**: free of medical conditions affecting metabolism, >2% above their age- and sex-specific body fat mass  **Exclusion criteria:**  attended the Weigh to Stay program within the past year, were pregnant or 180 days postpartum, were likely to leave military service within 1 year, or were deploying within 6 months | **Group:** Diet & education  Weight to stay alone (1) or meal replacement program in addition (2), 1: 3 educational sessions within a 2-week period, 2: Slim-Fast Plan | **Changes all volunteers:** Weight (kg): Weigh to Stay 98.3±14.3 Change prä/post: 0.81±2.6 (p<0.05) ,meal replacers: 96.1±16.1 change: 2.0±3.2 (p<0.01) , p between both groups: p<0.05 BMI Weight to stay: 32.0± 3.0 Change prä/post: 1.1± 1.1 Meal replacers: 31.9 ±3 change prä/post: 1.2 ±1  significant change: body composition, fasting blood lipid concentrations, energy intake, body fat in the groups | PEDro 5/11 |
| Author: Smith et al  Year: 2012  Region: USA  Title: Efficacy of orlistat 60 mg on weight loss and body fat mass in US Army soldiers [^9^].  Study Type: RCT | **overall:** n= 435 **male:** n= 325 **female:** n=110 **completed:** 22 Placebo, 35 Orlistat  **Baseline characteristics**:  **Inclusion criteria**: >2% above their percent body fat standard (6) and free of medical conditions affecting metabolism, appetite, or capacity for physical activity,  **Exclusion criteria:**  attended the AWCP Weigh-to-Stay sessions within the past year, previously used orlistat 60 mg, were pregnant or 180 days postpartum, were leaving military service within 1 year, or were reporting for duty outside of the United States within 6 months. | **Group:** medication & education  Orlistat 60mg 3xd or Placebo + Weigh to Stay Protocol (3 education sessions) | **Changes baseline to month 6:** **PPA** (placebo n=22, orlistat n=35):  no significant difference in body weight, fat-free mass, and fat mass between groups Weight (kg): placebo: 102.4 ±16.2 (pre) 99.4± 15.4(post) 3.0 ±5.2(change), orlistat: 97.8± 15.6(pre) 94.7± 17.2(post) 3.1± 4.7 (change) (both significant reduction in the group) BMI: placebo 33.5 ± 3.8(pre) 32.5 ± 3.7(post) 1.0 ±1.7 (change), orlistat: 33.2 ±3.1 (pre) 32.1 ±3.7 (post) 1.1 ± 1.7 (change) (both significant in group) change in percent body fat: orlistat vs placebo mean difference 1.7% 2.0%; P 0.01  **ITT** :(N 252; placebo n 124, orlistat n 128),  orlistat: percent body fat 0.8%± 2.0% p<0.01, fat mass: 0.7±2.4 kg, P 0.05  Success in meeting body fat standards was higher for the orlistat group vs the placebo group (n 7 and n 0, respectively; P 0.01).   Blood pressure and blood chemistries were within normal ranges at baseline and no changes over time were detected within or between groups  **Attrition rate:** 86% | PEDro 6/11 |
| Author: LaFountain et al  Year: 2019  Region: Finland  Title: Extended Ketogenic Diet and Physical Training Intervention in Military Personnel[^10^].  Study Type: CCT | **overall:** n= 34, 5 withdrew --> n=29  **male:** n= 25 **female:** n= 4 KD=17, MD 17 (KD 15, MD 14)  **Baseline characteristics**: BMI MD: 24.9±2.4, KD: 27.9±2.9, p=0.005 -> significant difference before intervention Weight (kg): MD: 79.8 ± 5.5 KD: 85.7 ± 7.8 p=0.022 Age (years): MD: 24.6 ± 9.0 KD: 27.4 ± 6.8 p: 0.345  **Exclusion criteria:**  previous experience with Ketogenic diet, >50years, met specific health criteria (endocrine dysfunction, hormonal imbalances, medication, injuries, allergies, cardiovascular disease, non-smokers), or could not exercise safely | **Group:** Mixed Diet/Exercise  Ketogenic or mixed diet group, KD: frequent coaching, They were provided unlimited amounts of frozen, pre-cooked meals (Quest Nutrition, El Segundo, CA) and grocery supplies for the duration of the intervention, Carbohydrate and protein intakes were initially limited to 25 and 90 g/d, KD participants were provided with a ketone/glucose monitor (Precision Xtra, Abbot, Illinois) for daily measurements of capillary glucose and βHB.  MD: The MD group participants maintained their habitual diet with a minimum consumption of ~40% carbohydrate.  physical training intervention in a 12wk supervised progressive resistance training programm 2d/wk | **BMI:** KD vs MD (−7.7 kg vs 0.1 kg; p < 0.001) from baseline to post intervention,  **Body fat percentage:** KD vs MD (−5.1 vs −0.7%; p < 0.001),  Mean visceral fat volume: KD vs MD (−561.3 vs −1.9 cc; p < 0.001) Whole-body fat mass: KD vs MD (−5.9 vs −0.6 kg; p < 0.001)  There were no differences at baseline or in response to the intervention between groups in a majority of strength and power measures of anaerobic performance | NHLBI 10/14 |
| Author: Stewart et al  Year: 2011  Region: USA  Title: H.E.A.L.T.H.: efficacy of an internet/population-based behavioral weight management program for the U.S. Army[^11^].  Study Type: CCT | **overall (military):** n= 2417 **male:** n= 1662 **female**: n= 755 military and civilians (einzelne Darstellung),   **Inclusion criteria**: must meet body weight and body fat standards from Army weight control program.   **Baseline characteristics**: Age Women (755) 30.9 (8.5) , Men (1662) 32.3 (8.1) BMI Women (677) 26.8 (4.0), Men (1473) 28.9 (3.7)  **Exclusion criteria**:  Not reported | **Group:** Life Style intervention  1: Internet-based weight management programm,2: promotion program, access 3 years (25 month active) | **self-reported changes in body weight:** body weight loss >5% : Group 1: 6% (from 181), Group 2: 12% (from212), 3: 33% (from 54), total: 12%,  (12% of participants lost ≥5% at some point during the study, and 10% of participants lost ≥5% as their last weight entry) o significant change between low users and high users in BMI or Age correlation between Web site visits and weight change (%) at the end of the study was found to be significant (r = -.21, p < .0001) | NHLBI 8/14 |
| Author: Webber et al  ^12^  Year: 2012  Region: USA  Title: Indicators of sequential fitness assessment failures for Travis Air Force Base airmen who attend the Be Well Course [^12^].  Study Type: cohort study | **overall:** n= 276 **male:** n= 211 **female:** n= 65  **Inclusion criteria**: military personnel who failed the Fitness Assessment,    **Baseline characteristics**: age 19-51 years (29± 6.9),  BMI: 27.9 +-3.9 (males), 25.2 +-3.3 females,  AC (in):  time between FAs in average: 75days (4-201),  **Exclusion criteria:**  deployment, pregnancy, commander exemption | **Group:** Life Style intervention  a behavioural modification class during 1 days after failure, 4 hour course | **BMI (kg/m2)**  men: FA1: 27.9 ± 3.9 FA2: 27.7 ± 3.8 p=0.005;  women: FA1 25.2 ± 3.3 FA2: 25.0 ± 3.4 p=0.087  **Waist circumference (Inches)**  men: FA1 35.4 ± 3.3 FA2 35.1 ± 3.3 p<0.001  women: FA1 28.5 ± 2.8 FA2 28.4 ± 2.8 p= 0.766 | NHLBI 10/14 |
| Author: Brandes, Mirko  Year: 2007  Region: Germany  Title: Bestimmung des Aktivitätsniveaus adipöser Soldaten im Rahmen des Adipositas-Interventionsprogramms der Bundeswehr [^13^].  Study Type: cohort study | **overall:** n= 23 (12 from 9, and 11 from 10) with SAM  Participants of the obesity intervention program of the Bundeswehr in Warendorf, AIP course 9 and 10, the others each as control group,  **Exclusion criteria:**  Not reported | **Group:** Exercise  Step activity monitor (SAM) | significant parameters: Muscle mass p= 0.01 Fat mass p= 0.04 waist circumference p=0.01 Cholesterol: p=0.01 LDL: p=0.01  not-significant: BMI, Weight, HDL, power  Correlation between weight reduction and average number of gait cycles per year (r=-0.4), not significant circumference r=-0.3 (n.s.) BMI r=-0.3 n.s.  ST09 proband: Weight: -0.81kg, BMI -0.22, Circumference: -2.5cm ST09 control: Weight: -3.52kg, BMI -1.04, Circumference: -6.28cm ST010 proband: Weight: -4.3kg, BMI -1.3, Circumference: 3.7cm ST10 control: Weight: -4.68kg, BMI -1.53, Circumference: -5.75cm | NHLBI 10/14 |
| Author: Sammito, Stefan  Year: 2012  Region: Germany  Title: Das Adipositas-Interventionsprogramm der Bundeswehr [^13^].  Study Type: cohort study | **overall**: n= 1090  n=625 ambulant, n= 425 course  **Inclusion criteria:**  BMI >30/27 and 2 cvRF, Remaining service time >2 years,  n=1090,   **Baseline characteristics:** Weight (kg) total: 109,3 ± 15,6 ambulant: 109,8 ± 16,5 course: 108,6 ± 14,1 BMI (kg/m²) total: 33,8 ± 4,0 ambulant: 33,8 ± 4,2 course: 33,7 ± 3,6 Age (years): total: 41,2 ± 8,9 ambulant: 40,0 ± 9,4 course: 43,0 ± 7,3 Waist circ. (cm): total: 112,3 ± 10,6 ambulant: 112,0 ± 11,2 course: 112,7 ± 9,8  **Exclusion criteria:**  Not reported | **Group:** life style intervention  2-year life style intervention  sports and medical training  nutritional advice | Weight controls, bioimpedance and laboratory,  statistically significant improvement in weight and absolute power on the cycle ergometer,  mean weight loss after 12 months: ambulant: 7kg, course: 4.4kg;  power: ambulant: 31.6 watts, course: 16.2kg,  no successful weight reduction (>50% weight reduction of at least 5%, at least 20 % of 10%), after ITT (n=313): 28.7% have lost 5%, (n=114) 10.5% have reduced 10%  **Attrition Rate:** 60% (12 months) | NHLBI 06/14 |
| Author: Sammito, Stefan  Year: 2016  Region: Germany  Title: Obesity Intervention During a Work Health Promotion [^14^].  Study Type: cohort study | **overall:** n= 665 **male:** n= 630 **female:** n=35  **Inclusion criteria**: soldier with a BMI of 30 kg/m2 or more alone or a BMI of 27.5 kg/m2 or more and an additional cardiovascular risk factor (eg, smoking or high blood pressure), service for minimum 2 more years,   **Baseline characteristics:** Overweight: 94 (14,4%), Obesity (561 (85,6%),  BMI: 33.8± 4.2kg/m²,  Age: 40± 9,4 bodyweight 109.8 ± 16.5 kg  **Exclusion criteria**:  Not reported | **Group:** life style educaton (⬄PA+diet)  Obesity Intervention Programme:  Biometrics and blood analysis, bicycle ergometer with ECG and lactate to make an individualized advice for more physical activity in daily life + diet analysed by a nutritionist with recommendation on basis of the German Society pf Nutrition, ambulant Version | **Parameters at beginning and the follow-up dates: *P 0.05; **P 0.01; ***P 0.001.**  **Bodyweight:**  kg 0m: 109.8± 16.5 3m: 107.9± 1.2*** 6m:107.1± 16.5*** 9m: 106.8±16.5*** 12m: 106.7± 16.6*** 18m: 106.7 ±16.5*** 24m: 106.7± 16.6*** p= 0.001  **BMI:**  0m: < 33.8± 4.2 3m: 33.2± 4.2*** 6m: 33.0± 4.3*** 9m: 32.9± 4.3*** 12m:32.9± 4.4*** 18m:32.9± 4.4*** 24m: 32.9± 4.4*** p=0.001  **Waist circumference (cm)** 0m: 112.0± 11.2 3m: 110.3± 11.3*** 6m: 109.4 ± 11.5*** 9m:109.0± 11.7*** 12m:108.9 ± 11.7*** 18m: 109.0 ± 11.7*** 24m: 108.9 ± 11.8*** p= <0.001 | NHLBI 10/14 |
| Author: Sammito, Stefan  Year: 2016  Region: Germany  Title: Results of a course based obesity interven^14^tion program during work [].  Study Type: cohort study | **overall:** n= 334 **male:** n= 329 **female:** n= 5  **Baseline characteristics**: Age. 43.5 ± 6.9 years,  BMI of 33.4 ± 3.6 kg/m2 Body Weight: 107.4 ± 13.8  **Inclusion criteria:** Participants of obesity intervention program (BMI>30 oder BMI>27,5+cvRF), 2 years left  **Exclusion criteria**:  none | **Group:** life style ⬄ advice PA  3 weeks in Warendorf (medical check, physical fitness), one week after 12 months and one week after 24 months, follow-up: 6,12,18,24 months, individual advice for more activity in everyday life and for optimal sporting activity with heart rate zones, course version | **Parameters at beginning and the follow-up dates: *P 0.05; **P 0.01; ***P 0.001.**  **Bodyweight [kg]**  6m: –4.8 ± 5.2∗∗∗ 12m: –3.7 ± 6.2∗∗∗ 18m: –3.9 ± 6.2∗∗∗ 24m: –3.4 ± 6.6∗∗∗  **BMI [kg/m2]**  6m: –1.5 ± 1.6∗∗∗ 12m: –1.1 ± 1.9∗∗∗ 18m: –1.2 ± 1.9∗∗∗ 24m: –1.0 ± 2.0∗∗∗  after 24months significant difference in: Bodyweight, BMI, Waist circumference, physical capacity, Blood pressure, AST, Cholesterol, HDL, LDL, Uric acid, HbA1c,  not significant: ALT, GGT, Triglyceride | NHLBI 09/14 |
| Author: Vantarakis et al  Year: 2022  Region: Greek  Title: The Effects of Exercise During a 10-Week Basic Military Training Program on the Physical Fitness and the Body Composition of the Greek Naval Cadets [^14^].  Study Type: cohort study | **overall:** n= 185 **male:** n=153  **female:** n= 32  **Baseline characteristics**: age: 18.4 ± 0.7 years, height: 1.77 ± 6.7 m, BM: 72.6 ± 9.1 kg),  male (age: 18.3 ± 0.6 years, height: 1.79 ± 5.8 cm, BM: 75.3 ± 7.6 kg)  female (age: 18.6 ± 0.9 years, height: 1.68 ± 2.8 cm, BM: 59.9 ± 3.1 kg),   **Inclusion criteria:** considered fit, being non-smokers, free of pharmacological treatments, no previous experience on military tasks and the past 2 years presented athletic training for 1.2 ±1.1 hours per week  **Exclusion criteria:**  Not reported | **Group:** Exercise  Basic military training (BMT) 10-week, each year for 4 years in the BMT period performed 94 training sessions lasting 50-60 minutes five times a week | first measurement was performed on days 1–3 in the first week, second measurement was performed immediately after the end of the 10th week.   BM (kg)  Men pre 75.3 ± 7.6 Men Post 73.3 ± 6.7  Women pre 59.9 ± 3.1 women post 58.6 ± 2.9  total pre72.6 ± 9.1 total post 70.8 ± 8.4   BMI (kg × m−2)  Men pre:23.6 ± 2.7 Men post: 22.9 ± 2.5  Women pre: 21.4 ± 0.9 Women post: 20.9 ± 0.9  Total Pre: 23.2 ± 2.6 Total post: 22.6 ± 2.4   %Bodyfat  men pre: 10.8 ± 3.7 men post: 9.2 ± 2.9  women pre: 19.7 ± 3.9 women post: 19.3 ± 2.9  total pre. 12.4 ± 5.0 total post: 11.0 ± 4.8  BMI (t184 = 17.681, P < .01) decreased significantly by 2.6% with the corresponding reductions in men (t152 = 16.046, P < .01) by 3% and in women (t31 = 9.224, P < .01) by 2.3%,   The percentage reduction in %BF in all cadets reached 11.3% (t184 = 8.134, P < .01), | NHLBI 09/14 |
| Author: Bowles, Stephan  Year: 2006  Region: USA  Title: The LIFE program: a wellness approach to weight loss.  Study Type: cohort study | **overall:** n= 93, 53 completed follow up,  **male**: n= 55 **female:** n= 38  **Inclusion criteria**: military personnel, overweight or obese, 12 months remaining on location, emotionally stable,   **Baseline characteristics**: Men (n 35):  Weight (pounds) mean 216.34 SD 25.97  BMI mean 31.927 SD 2.66   Women (n 18) : Weight (pounds) mean: 183.44 SD: 30.25  BMI mean 29.786 SD: 3.50  **Exclusion criteria:**  Not reported | **Group:** Life Style intervention  LIFE (lifestyle change, individual readiness, fitness excellence, eating healthy) wellness program, a psychology directed, multidisciplinary team of health care providers offering an intensive lifestyle change treatment approach. | Men (n 35):  Weight (pounds) 205.69± 24.31 (t-Score) 7.047 p<0.001;  BMI 30.37± 2.65 (t-Score)6.971 p< 0.001   Women (n 18):  Weight (pounds) 169.44± 27.62 (t-Score) 5.833 p<0.001;  BMI 27.485± 2.97 (t-Score) 5.880 p<0.001 | NHLBI 08/14 |
| Author: Hunter, Christine  Year: 2008  Region: USA  Title: Weight management using the internet a randomized controlled trial.  Study Type: RCT | **overall:** n= 446 **male:** n= 222 **female**: n= 224  **Baseline characteristics**: Age (years) BIT: 33.5 ±7.4 Usual care: 34.4 ±7.2  **Exclusion criteria:**  lost more than 10 pounds in the previous 3 months; used prescription or over-the-counter weight-loss medications in the previous 6 months; had any physical activity restrictions; had a history of myocardial infarction, stroke, or cancer in the last 5 years; reported diabetes, angina, or thyroid difficulties; or had orthopaedic or joint problems that would prohibit exercise. Women: pregnant, breast-feeding, plans to become pregnant in the next year. | **Group:** Life style education program | **Outcomes to 6 months** BIT:  Weight (kg) pre-test: 87.4 ±15.6 post-test: 85.5± 15.8 Change: 1.3± 4.1  BMI (kg/m2) pre-test: 29.4 ± 3.0 post-test: 28.8 ± 3.3 change: 0.5 ± 1.4 Waist circumference (cm) pre-test: 94.5± 11.0 post-test: 92.2± 11.6 change: 2.1± 4.3  Usual Care:  Weight (kg) pre-Test: 86.6 ±14.7 post-test: 87.4± 14.7 change: 0.6± 3.4  BMI: pre-test: 29.3 ± 3.0 post-test: 29.4 ±3.0 change: 0.2 ±1.1   Waist circumference (cm): pre-test: 94.2± 10.9 post-test: 93.4 ±12.8 change: 0.4± 3.8   Weight loss, BMI and percentage body fat: BTI to usual care p<0.001 | PEDro 7/11 |
| Author: Cederberg, Henna  Year: 2011  Region: Finland  Title: Exercise during military training improves cardiovascular risk factors in young men.  Study Type: CCT | **overall:** n= 1467 were invited (starting in the Brigade), 1160 (79% enrolled) **male:** n= 1467 **female:** n= 0  **Baseline characteristics**: mean age of 19.3 years (range 19–28) Weight (kg) (mean, range) n= 1070, 75.1 (47.2–140.0) BMI (kg/m2) (mean, range) n= 1070, 23.9 (16.3–46.0) Waist circumference (cm) n= 1056, 81.6 (10.2)  **Exclusion criteria:**  Not reported | **Group:** Exercise  intensive exercise intervention, 6-12months follow up | Weight (kg) (mean, range) n= 1070  Baseline: 75.1 (47.2–140.0) Change: −0.42 (5.19) p= 0.008   BMI (kg/m2) (mean, range) n= 1070  Baseline: 23.9 (16.3–46.0) Change: −0.30 (1.69)p <0.001   Waist circumference (cm) n= 1056  Baseline: 81.6 (10.2) Change: 0.10 (5.8) p=0.589   Fat mass (kg) n= 1030  Baseline: 13.4 (9.2) Change: −1.3 (4.7) p<0.001   Fat % n= 1030  Baseline: 16.7 (8.2) Change: −1.1 (4.6) p<0.001  significant change in: systolic blood pressure, HDL, cholesterol | NHLBI 11/14 |
| Author: Parastouei, Karim  Year: 2022  Region: Iran  Title: Effects of symbiotic supplementation on the components of metabolic syndrome in military personnel: a double-blind randomised controlled trial  Study Type: RCT | **overall:** n= 60 **male:** n= 35 **female:** n=25 **Inclusion**: personnel with metabolic syndrome, age 20-50  **Baseline Characteristics: 30suppl., 30placebo** Age: 42.33 ± 1.49(Supplement), 40.6 ± 1.13 (Placebo), p0.3 BMI: 32.51 ± 0.87 (Suppl.), 31.64 ± 0.79 (Placebo), p0.46 Waist circumference (cm): 106.93 ± 1.9 (suppl.), 104.53 ± 1.44 (placebo), p0.32  **Exclusion criteria:**  history of CVDs, liver and kidney failure, alcohol abuse, smoking, postmenopausal women, | **Group:** medication (suppl.)  supplements (FamiLact, Lactobaccilus and other) for **8 weeks**, once a week phone call by trained nutritionist | significant impact on BMI after adjusting the effect of intervention for the pre-intervention values of outcome variables | PEDro 8/11 |
